# Carbonic Anhydrases and Metabolism

**DOI:** 10.3390/metabo8020025

**Published:** 2018-03-21

**Authors:** Claudiu T. Supuran

**Affiliations:** Dipartimento Neurofarba, Sezione di Scienze Farmaceutiche, Laboratorio di Chimica Bioinorganica, Università degli Studi di Firenze, Polo Scientifico, Via U. Schiff 6, Sesto Fiorentino, 50019 Florence, Italy; claudiu.supuran@unifi.it

**Keywords:** carbonic anhydrase, hypoxic tumor, metabolism, carboxylation, bicarbonate, pH regulation, antitumor agent, sulfonamide, bacterial enzymes

## Abstract

Although the role of carbonic anhydrases (CAs, EC 4.2.1.1) in metabolism is well-established, pharmacological applications of this phenomenon started to be considered only recently. In organisms all over the phylogenetic tree, the seven CA genetic families known to date are involved in biosynthetic processes and pH modulation, which may influence metabolism in multiple ways, with both processes being amenable to pharmacologic intervention. CA inhibitors possess antiobesity action directly by inhibiting lipogenesis, whereas the hypoxic tumor metabolism is highly controlled by the transmembrane isoforms CA IX and XII, which contribute to the acidic extracellular environment of tumors and supply bicarbonate for their high proliferation rates. Many of the articles from this special issue deal with the role of cancer CAs in tumor metabolism and how these phenomena can be used for designing innovative antitumor therapies/imaging agents. The metabolic roles of CAs in bacteria and algae are also discussed.

Carbonic anhydrases (CAs, EC 4.2.1.1) are a superfamily of metalloenzymes present in all life kingdoms, as they equilibrate the reaction between three simple but essential chemical species: CO_2_, bicarbonate, and protons [1,2,3,4,5,6]. Although discovered 85 years ago, these enzymes are still extensively investigated due to the biomedical application of their inhibitors [7,8,9,10,11,12] and activators [13] but also because they are an extraordinary example of convergent evolution, with seven genetically distinct CA families that evolved independently in Bacteria, Archaea, and Eukarya, the α-, β-, γ-, δ-, ζ-, η-, and θ-CAs [2,4,5,14,15,16]. CAs are also among the most efficient enzymes known in nature, probably due to the fact that uncatalyzed CO_2_ hydration is a very slow process at neutral pH, and the physiologic demands for its conversion to ionic, soluble species (i.e., bicarbonate and protons) are very high [1,2,3,4,5,6]. Indeed, CO_2_ is generated in most metabolic oxidative processes, and being a gas, it must be converted to soluble products quickly and efficiently. Otherwise, it would tend to accumulate and provoke damage to cells and other organelles in the gaseous state without such an efficient hydration catalyst as the CAs [2,6,7,8].

Inhibition of the CAs has pharmacologic applications in many fields, such as diuretics [9], antiglaucoma [10], anticonvulsant [7,8,11], antiobesity [11], and anticancer agents/diagnostic tools [1,2,12], but it is also emerging for designing anti-infectives, i.e., antifungal, antibacterial, and antiprotozoan agents with a novel mechanism of action [4,5,8,17,18]. For a long period it has been considered that the pharmacologic effects of CA inhibition or activation are mainly due to effects on pH regulation in cells or tissues where the enzymes are present [1]. Although these phenomena are undoubtedly relevant and take place in most organisms/tissues/cells where these ubiquitous enzymes are found, a lot of recent evidence points to the fact that CAs are true metabolic enzymes at least for two different reasons: (i) due to their direct participation in carboxylating reactions which provide bicarbonate and/or CO_2_ to carboxylating enzymes, such as pyruvate carboxylase, acetyl-coenzyme A carboxylase [19,20], phosphoenolpyruvate carboxylase [21], and ribulose-1, 5-bisphosphate carboxylase oxygenase (RUBISCO) [22,23]; and (ii) due to the role that pH itself has on many metabolic reactions, with pH differences as low as 0.1 unit leading to the complete blockade of crucial reactions and thus of entire metabolic pathways [1,2,3]. For these reasons, the CAs may be considered as important checkpoint enzymes for relevant physiologic processes connected to a host of metabolic pathways, in all types of organisms, from bacteria and archaea [24,25] to algae, plants [26], and other eukaryotes (starting with the simple ones, yeasts and protozoa, and ending with the complex ones, including vertebrates) [1,2,3,4,5,6,7].

The metabolic reactions with which CA activity interference has been mostly studied include de novo glucogenesis, urea biosynthesis, and lipogenesis in animals [1,2,3,4,5,6,7,19,20] as well as the initial steps of the photosynthetic process in some bacteria, algae, and plants, due to the role that CAs have in providing bicarbonate (through a carbon-concentrating mechanism) to RUBISCO [22,23,26]. In tumors, these metabolic processes are even more complex, as it has been shown that not only do the protons produced by CO_2_ hydration contribute to extracellular acidification, typical of most cancers [1,2,12,27], but the bicarbonate is thereafter used as a C_1_ carbon source for biosynthetic reactions that convert it into organic compounds (the so-called “organication”), which supplies cancer cells with intermediates useful for sustaining their high proliferation rates [28]. Inhibition of the various CA isoforms/CA enzyme classes involved in these phenomena, mainly with sulfonamides, the most widely used class of CA inhibitors [29,30,31,32,33,34,35,36], has important physiological consequences which motivate their use as pharmacological agents as mentioned earlier. Whereas the use of carbonic anhydrases inhibitors (CAIs) as diuretics and antiglaucoma agents has been well-established for decades [1,7,8,9,10], their applications as antiepileptics and antiobesity drugs is more recent [1,7,11,13,20], and only the last decade has seen important advances which have validated CAs as antitumor drug targets [3,12,27,35]. Thus, it is not unexpected that five papers of the special issue deal with the connections between tumor-associated CAs and tumors and the development of new anticancer agents based on them. The first such paper [27] reviews the role that hypoxia has in triggering a diverse metabolism to cancer cells, all of which are orchestrated by the transcription factor hypoxia-inducible factor 1α (HIF-1α), which in the end leads to the overexpression of at least two CA isoforms, which are scarcely present in normal tissues, CA IX and XII. Inhibition of these two enzymes with sulfonamides or coumarins was shown to impair the growth of the primary tumors and metastases and to reduce the population of cancer stem cells, leading thus to a complex and beneficial anticancer action for this class of enzyme inhibitors [27,37,38,39,40]. The paper of McDonald et al. [37] discusses CA-mediated regulation of pH together with the recent proteome-wide analyses that have revealed the presence of a complex CA IX interactome in cancer cells, which has multiple roles in metabolite transport and tumor cell migration and invasion. In both these papers [27,37], the various aspects of the development of the first antitumor agent from this class that reached clinical development, SLC-0111, are discussed, considering the fact that these two groups are the discoverers of this new drug.

Mboge et al. [38] consider not only CA IX and XII, the most investigated proteins of this family connected to cancers, but all CA isoforms from mammals and their possible role in tumors as potential targets for cancer therapy. This interesting paper proposes thus that in addition to CA IX/XII, other isoforms, such as the mitochondrial ones CA VA/VB, or some of the cytosolic CAs (isoforms I, II, VII, and XIII) might become anticancer drug targets sooner or later [38].

Ward et al. [39] discuss the various classes and types of CA IX inhibitors which were investigated in detail in various models and systems, together with the fact that this class of pharmacological agents may enhance the effects of anti-angiogenic drugs or chemotherapy agents by different mechanisms that are poorly understood at this moment. Work from their laboratories also showed that CA IX interacts with several of the signaling pathways involved in the cellular response to radiation, suggesting that pH-independent mechanisms may also be important for the role that CA IX inhibitors in combination with radiations have in slowing down tumor progression [39,41].

Iessi et al. [40] discuss the possibility of combining CA IX/XII inhibitors with inhibitors of other proton exchangers and transporters present in tumor cells, such as V-ATPase, Na⁺/H⁺ exchangers (NHE), and monocarboxylate transporters (MCTs). In fact, recent work suggests that a strong synergistic effect is observed when combining CAIs with proton pump inhibitors of the lansoprazole/omeprazole type [40,42]. Furthermore, the drug delivery of anticancer agents by means of exosomes (natural extracellular nanovesicles), which exploit tumor acidity as a molecular engine, has been proposed by the same group [43].

With the exception of CAs and the cancer metabolism connection, with various aspects discussed in the papers mentioned above [27,37,38,39,40], Parkkila’s group [26] thoroughly reviewed the roles that different CAs have in the algal model organism *Chlamydomonas reinhardtii*. It is in fact well-known that photosynthetic organisms contain six evolutionarily different classes of CAs, the α-CAs, β-CAs, γ-CAs, δ-CAs, ζ-CAs, and θ-CAs, and many of them possess more than one isoform in the same organism [26]. *Chlamydomonas reinhardtii* contains 15 CAs belonging to three gene families: three α-CAs, nine β-CAs, and three γ-CAs, with quite a different subcellular localization. The review presents the known metabolic roles that some of these enzymes have in the carbon-concentrating mechanism which provides bicarbonate to RUBISCO for photosynthesis, but also predicts functions for some of these CAs for which precise metabolic roles are yet to be discovered [26].

Bacteria also possess CAs belonging to three diverse genetic families, the α-CAs, β-CAs, and γ-CAs [4,5,25]. Supuran and Capasso [25] reviewed the roles that these enzymes have in these organisms, predominantly considering pathogenic bacteria, such as *Escherichia coli*, *Vibrio cholerae*, *Brucella suis*, *Helicobacter pylori, Porphyromonas gingivalis*, *Mycobacterium tuberculosis*, and *Burkholderia pseudomallei*. For many of these bacteria, one or more CAs belonging to the three classes were cloned, characterized, and investigated for their inhibition with the main classes of CAIs in the search for antibacterial agents with a new mechanism of action that is free of the drug-resistance problems of currently used antibiotics [25]. Although this field is still in its infancy, substantial progress has been achieved ultimately in understanding the roles that these enzymes have in the life cycle and virulence of many pathogens provoking serious diseases.

Overall, this interesting special issue of *Metabolites* affords a series of interesting reviews which show the multitude of aspects connecting simple enzymes, such as the CAs to metabolic processes, in all types of organisms. They may afford both a better understanding of fundamental processes, such as carbon capture in photosynthesis, tumorigenesis, and the role of pH in metabolism, but also lead to the development of novel therapeutic strategies in areas such as oncology and anti-infective agents.
